# The 2021 western North America heat wave among the most extreme events ever recorded globally

**DOI:** 10.1126/sciadv.abm6860

**Published:** 2022-05-04

**Authors:** Vikki Thompson, Alan T. Kennedy-Asser, Emily Vosper, Y. T. Eunice Lo, Chris Huntingford, Oliver Andrews, Matthew Collins, Gabrielle C. Hegerl, Dann Mitchell

**Affiliations:** 1 School of Geographical Sciences, University of Bristol, Bristol, UK.; 2 UK Centre for Ecology & Hydrology, Wallingford, UK.; 3 College of Engineering, Mathematics, and Physical Sciences, University of Exeter, Exeter, UK.; 4 School of Geosciences, University of Edinburgh, Edinburgh, UK.

## Abstract

In June 2021, western North America experienced a record-breaking heat wave outside the distribution of previously observed temperatures. While it is clear that the event was extreme, it is not obvious whether other areas in the world have also experienced events so far outside their natural variability. Using a novel assessment of heat extremes, we investigate how extreme this event was in the global context. Characterizing the relative intensity of an event as the number of standard deviations from the mean, the western North America heat wave is remarkable, coming in at over four standard deviations. Throughout the globe, where we have reliable data, only five other heat waves were found to be more extreme since 1960. We find that in both reanalyses and climate projections, the statistical distribution of extremes increases through time, in line with the distribution mean shift due to climate change. Regions that, by chance, have not had a recent extreme heat wave may be less prepared for potentially imminent events.

## Teaser

Heat extremes greater than the June 2021 western North America event have previously occurred elsewhere.

## INTRODUCTION

Heat extremes are a natural part of our climate system but are getting hotter and longer in duration because of human-induced climate change (*1*). Heat extremes pose a threat to human and ecological health (*2*, *3*), and the chance of extreme heat events has increased in most regions around the world (*4*–*6*). Excess mortality due to extreme heat is well documented, with an average of 6 heat-related deaths per 100,000 residents each year in North America estimated for 2000–2019 (*7*). Heat impacts are magnified in cities, and with nearly 70% of the worlds’ population expected to live in cities by 2050, the risks posed by extreme heat events will also increase (*8*, *9*).

In June 2021, a record-breaking heat wave affected North America. The Canadian town of Lytton broke local temperature records by 4.6°C, setting a national temperature record of 49.6°C (*10*). The temperatures were so high that claims were made at the time that Earth system models (ESMs) could not reproduce them (*11*). Studies suggest that we need to be prepared for these “record shattering” events (*12*), but the question arises: Are the events we are observing becoming more extreme or just better reported? There may have been heat wave events as extreme in the past, which have gone relatively unnoticed due to occurring in regions with poor observations and low populations, where media centers have less of a presence.

With the alarm felt globally about the June 2021 North America extreme, there are concerns that the magnitude of extreme temperature events may be increasing faster than expected. Does the magnitude of change to heat events scale linearly with global warming (*13*)? Or do other factors, such as seasonality (*14*), soil moisture feedbacks (*15*), or sea surface temperature forcings (*16*), lead to an acceleration of likelihood of extreme events? Compound events, such as high temperature and low rainfall events, may increase impacts on humans (*17*).

The impacts of extreme temperature events vary depending on the climatological conditions, as well as other socioeconomic factors such as population density (*18*). To understand the implications of an extreme, we need to assess it compared to the climatology of both the time period and region where it occurs. If the usual conditions in a region have low variability, a smaller magnitude event (in terms of absolute temperature) may have a larger impact on, for example, the ecosystem. Humans may be used to a greater range of climatic conditions than flora and fauna (*19*). Given the increasing atmospheric temperatures, it is important to understand how many regions we expect to start seeing particularly extreme heat events. We must understand whether the rate of increase exceeds the adaptive rate of the region of interest. Conversely, we must be aware of the regions that, just by chance, have not seen a particularly extreme heat wave in recent decades—as those may be less prepared for these events. Evidence of this has been seen in western Europe, where heat-related mortality in a heat wave in 2006 was much lower than from an earlier heat wave in 2003 because of an increase in awareness (*20*).

ESMs allow us to estimate how the frequency of heat extremes will change as atmospheric greenhouse gases rise into the future. Recent simulations, bias-corrected against contemporary measurements, have been used to show that for much of Europe, the difference between 1.5° and 2.0°C of global warming could have a large impact on crossing local extreme temperature thresholds (*21*). Several studies have assessed the change in heat extremes in a future warmer world. Climate model simulations suggest a shift toward warmer extreme temperatures in the future (*22*), with increased variability in many regions (*23*). Historical gridded climate data provide benchmark measurements against which any ESM can be compared to understand its performance at estimating climatic statistics for the recent past. ESMs can also be used to assess whether past events could have been more extreme, using methods including extreme value analysis and creating large ensembles initialized from past events (*24*). Can ESMs reproduce extremes as large as seen this year in the current climate? In ESMs, how does the likelihood of recent extreme events reoccurring change into the future?

To understand in full recent extreme heat events, and in particular their context against background variability, requires a global assessment. Previous heat wave assessments have focused on particular regions and events, including the European heat waves of 2003 (*4*, *25*, *26*) and 2019 (*27*). Here, we provide a global characterization of the intensity of heat extremes over the record for which reanalysis products are available (1950–2021). The impact of a heat wave is highly dependent on the weather hazard, which we address, but other socioeconomic factors play a role, which we do not explicitly explore. We investigate the characteristics of the recent western North America heat wave and place the magnitude of this event in a global context. Using a large model ensemble, we assess the ability of an ESM to capture this event and project changes in the return period of similar heat extremes under future climate. Last, we assess global changes in the magnitude of heat extremes in both reanalyses and climate projections, relative to fixed and moving climatologies using two ESM large ensembles.

## RESULTS

### Western North America heat wave of June 2021

**Fig. 1.**
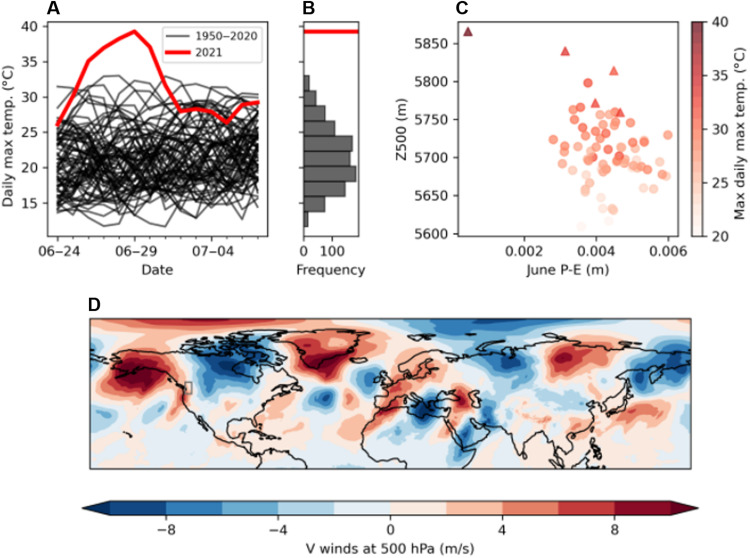
The meteorology of the western North America 2021 heat extreme. The synoptic pattern and statistics of the heat wave in the western North America for the last week of June and first week of July. (**A**) Time series plot of observed daily maximum temperatures for the last week of June and first week of July in the region 45°N to 52°N, 119°W to 123°W, from 1950 to present. The time series for individual years in 1950–2020 are shown in black, whereas that for 2021 is shown in red. (**B**) Distribution of the 1950–2020 temperatures shown in (A). The red line shows the highest maximum temperature in 2021, which happened on 29 June. (**C**) Scatter plot of geopotential height at 500 hPa (Z500) over the 2-week period 24 June to 7 July versus precipitation minus evaporation (P-E) over June, with both axes showing values averaged over the area of interest. Each dot represents 1 year between 1950 and 2021. The color of the dots indicates the maximum area–averaged daily maximum temperature over the corresponding 2-week period. Triangles indicate the five hottest events, with the triangle in the darkest red color indicating the 2021 event. (**D**) Northward component of wind at 500 hPa in the Northern Hemisphere, averaged over the same 2-week period in 2021; gray box over western North America indicates the region assessed: 45°N to 52°N, 119°W to 123°W.

To investigate the extreme temperatures of June 2021 in western North America, we assess data for the region 45°N to 52°N, 119°W to 123°W [Fig. 1 and (*28*); see Materials and Methods]. The daily maximum temperatures for the 2 weeks spanning the event, 24 June to 6 July, for all years 1950–2021 are examined (Fig. 1, A and B). The greatest regional average daily maximum temperature of 39.5°C was observed on 29 June 2021. For the preceding decade, the mean daily maximum temperature for the three hottest months is 23.4°C, with a standard deviation (SD) of 4.5°C. Therefore, the record June 2021 value is 3.6 SDs from the mean using this dataset. We note that the use of the preceding decade reference influences this value—as the mean daily maximum temperature has increased since 1950, this value would be higher using an earlier reference. We first consider this North American heat extreme in more detail and then assess whether similar magnitude events, defined by the number of SDs from the mean, have been seen elsewhere globally in the observational record.

We first investigate the drivers of the western North America heat wave. A combination of high atmospheric pressure and dry conditions may have caused the event (Fig. 1C). Drought conditions will lead to low soil moisture, which then creates a positive feedback, whereby more of the energy of the sun received at the land surface is converted into sensible heat rather than evaporating water from the soil (*31*), further warming the near-surface atmosphere. There is also evidence that lower soil moisture conditions act as a second positive feedback by prolonging anticyclonic conditions (*25*). The U.S. drought monitor shows severe to exceptional drought conditions across western North America in the lead up to the heat extreme, although this did not cover the full extent of the region, with northern parts showing wet anomalies (*28*, *31*). The pressure anomaly appears to be the main drier of the extreme heat, with soil moisture having a secondary effect. We show that some of the highest temperatures are seen in years with both a high pressure system over western North America and drier than normal conditions over the month running up to the event (Fig. 1C). The top two temperatures in late June are seen in years with high pressure over the region, and also dry conditions, represented by the difference between rainfall and evaporation (P-E).

The pattern of northward wind anomaly at 500 hPa (Fig. 1D) shows strong anomalies on either side of western North America, bringing warm tropical air northward into the region. A high pressure system dominated western North America, causing a “heat dome” effect (*10*), where the sustained near-stationary atmospheric pattern over the region forces air downward, leading to high surface air temperatures. This supports previous work, suggesting that the possible magnitude of extremes will vary depending on the particular large-scale circulation patterns (*29*). A planetary-scale wave pattern with wave number 5 is visible in the Northern Hemisphere (Fig. 1D).

### Modeling heat extremes in western North America

Extreme events, by their very definition, occur infrequently. This leads to model studies being particularly useful as multiple realizations of the real world can be performed. Ensembles of simulations enable the building of the statistical distribution of extreme events, allowing an assessment of the chance of occurrence of observed events. The computational demand of ESMs means that relatively few large ensembles are available. We use the large ensemble of the Canadian Earth System Model version 5 (CanESM5) from the Coupled Model Intercomparison Project Phase 6 (CMIP6) database (*32*, *33*) (see Materials and Methods), from which 50 ensemble members are available. We compare the full model ensemble to the ERA-5 data over western North America (45°N to 52°N, 119°W to 123°W), from 1981 to 2010. The model ensemble has a mean and SD similar to the reanalysis, with a model mean of 21.8°C compared to 22.6°C for the reanalysis and model SD of 5.1°C compared to 4.6°C (fig. S1). Despite this, when comparing individual ensemble members from the model, we find that the reanalysis lies outside the model distribution (fig. S2). In 1981–2010, the reanalysis mean is higher and SD lower than any of the individual ensemble members. Therefore, to assess the magnitude of model extremes, we compare to model climatology rather than to the reanalysis.

**Fig. 2.**
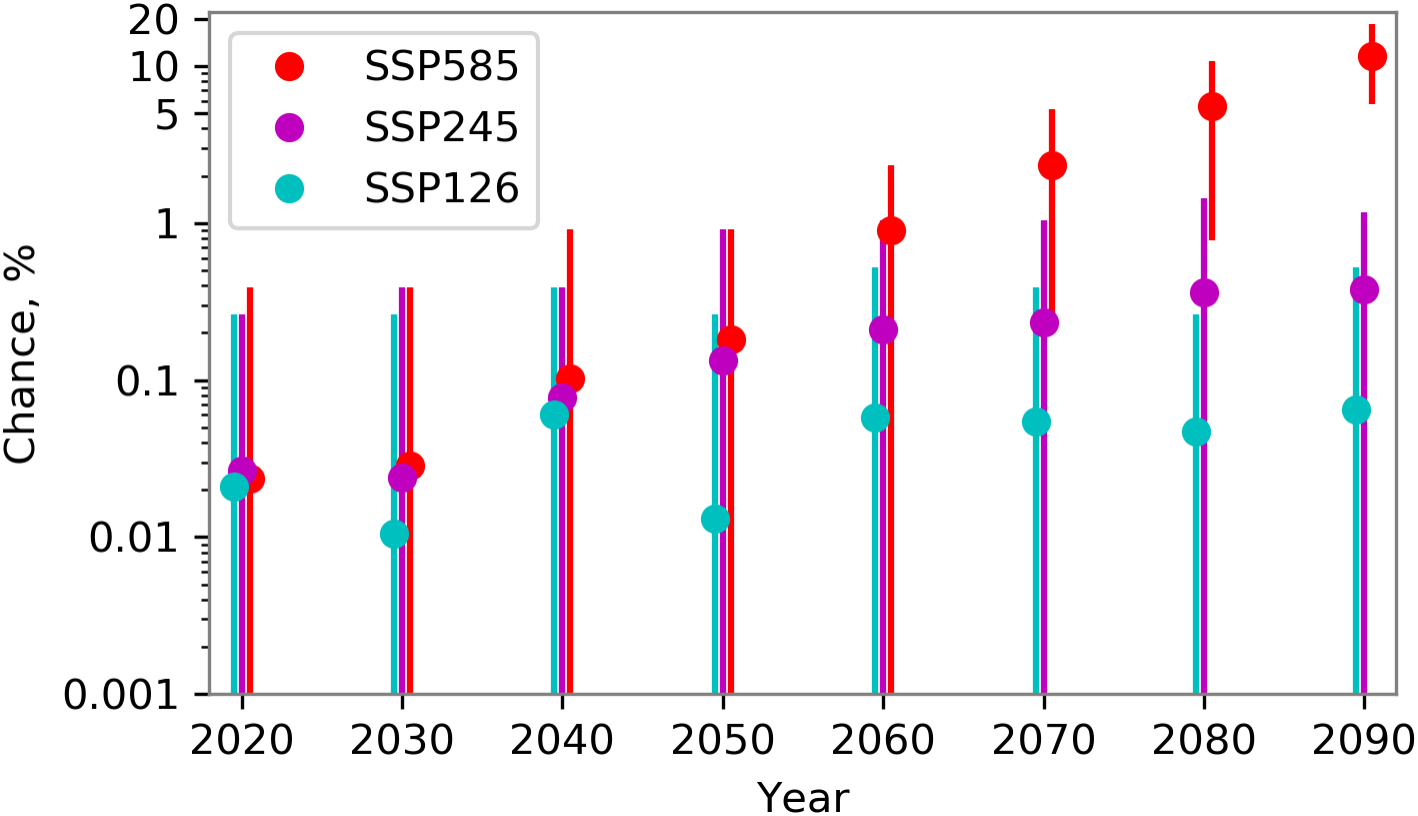
Projected change in the return period of the western North America heat extreme. The chance of exceeding the current record in any given summer day of a year in each decade. The current record used is 40.1°C, an event with the same magnitude as the June 2021 observed event for the model climatology, for the western North America region of 45°N to 52°N, 119°W to 123°W. Each decade is centered on the value on the *x* axis, (e.g., 2015–2024). Simulations are from the CanESM5, using three different future scenarios (as labeled) and 50 ensemble members for each scenario. Values are the chance of an event on any given day (JJA) per year. The vertical lines represent the uncertainty, calculated as the range from individual ensemble members.

Simulations of the current climate, 2015–2024, are assessed to evaluate the ability of the model to reproduce the observed temperatures of June 2021. The observed maximum, from the European Centre for Medium-Range Weather Forecasts (ECMWF) Reanalysis v5 (ERA5) data, is 39.5°C. The model is found to simulate extremes of 41.9°C in the region (fig. S1B). We find that, for the current climate, the chance of model extremes 3.6 model SDs from the model mean—the same relative magnitude as observed in June 2021—in any given summer [June-July-August (JJA)] day is 0.02% (Fig. 2). We can also use extreme value theory to assess the chance of this event, which gives a higher value of 0.4% (see Materials and Methods; fig. S3). Hence, for this model and current levels of atmospheric greenhouse gas concentrations, we can reproduce extreme events of similar magnitude to that observed in 2021.

The model projections run out to 2100, allowing us to investigate the chance of such an event in this particular region in the future climate. We use three different future scenarios, SSP126, SSP245, and SSP585 (see Materials and Methods) (*34*). Heat extremes greater than the observed record are found in every decade, from 2015 to 2024 onward. We show that by 2055–2064, the chance of an individual day experiencing higher temperatures than have been observed is 1-in-1000 to 1-in-75 year event, depending on the future scenario (Fig. 2). By 2085–2094, the chance increases to 1-in-6 years in the SSP585 future scenario.

### Global heat extremes in reanalysis

**Fig. 3.**
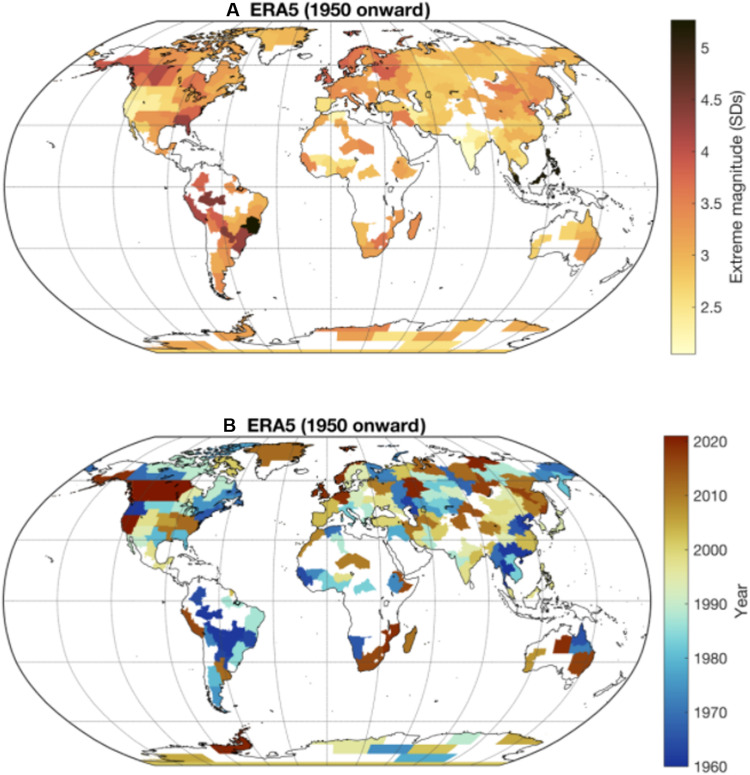
Global map of the most extreme observed heat extremes relative to the climate of that period. Data are taken from ERA5, January 1950 to August 2021. The values are expressed in terms of how many SDs away from the mean temperature the most extreme heat day was, assessed against a moving climatology so that the climate change signal is excluded (see Materials and Methods). The regions are taken from Stone, 2019 (*35*). (**A**) SD away from the mean of the greatest historic extreme in each region. (**B**) Year in which the greatest historic extreme occurred. Regions where there is poor agreement between ERA5 and JRA55 reanalyses are excluded.

To assess heat extremes globally, we use a dataset that divides land into ~0.5-million square meters (Mm^2^) regions that are specifically designed for climate impact studies (see Materials and Methods) (*35*). Reanalysis products assimilate a broad range of observations into an atmospheric circulation model to determine the best estimate of meteorological conditions on a regular spatial grid. We use reanalysis data to assess global extreme heat events from 1950 to present (*36*). We calculate a daily heat extremes index, of the number of SDs from the mean based on 10-year rolling climatologies, for each region (see Materials and Methods). Hence, our metric considers the local variability of the climate, relative to background warming. To gain confidence in our results, we use two reanalysis datasets—ERA5 and the Japanese 55-year Reanalysis (JRA55)—only including regions where the two agree (*37*, *38*). We compare this with station-based data for particular events of interest. The maximum daily values and the year of the most extreme event, based on our index, are mapped (Fig. 3, A and B). The western North America event of 2021 is apparent, spanning several regions, with a daily maximum over 4 SDs from the mean. This differs to the earlier result of 3.6 SDs due to the different regions used in the global assessment.

We tabulate the most extreme heat extremes, finding eight events that are consistent between the two reanalysis datasets. Many of the other regions that are identified as particularly extreme are surprising as they have not been widely reported, such as Southeast Asia in 1998 and Brazil in 1985 (Table 1). Some infamous events do not appear in this table, but that is in part because their notability comes from the overall impact of the event, rather than solely the variability of meteorology. The 2003 European heat wave is such an example, which still comes out as the largest event in France and Spain but does not feature in the top events. At 3.3 SDs above the mean, and with a relatively high SD (3.4°C), the absolute magnitude of the event is greater than many events that rank more highly with our method. More recent heat waves in France, of similar magnitudes, have had much lower human impacts due to adaptation resulting from changes in policy (*20*). The region and temporal scale used also influences the results. For example, for Switzerland, using mean summer temperatures, the 2003 heat wave was in excess of 5 SDs from the mean (*39*), but using daily temperatures, 2003 is not the most extreme. The Russian heat wave of 2010 does not show up because of the temporal scale we assess—assessments using longer time scales would highlight it. Despite record temperatures around the Mediterranean in July and August 2021, this event does not show because of our spatial averaging method.

**Table 1.** Top heat extremes that are consistent between datasets. Lists all events over 4 SDs (relative to the climate of the previous decade) from the mean in either ERA5 or JRA55, and consistent between the two datasets (see Materials and Methods). Because of the time periods covered by the reanalyses, only events from 1968 onward can be considered. The magnitude in terms of SD and absolute temperature, and the SD for both ERA-5 and JRA55 are included. Comments on the time scale and spatial extent of the events are included in the notes column.

**Region**	**Date**	**ERA5 (JRA55)** **Magnitude in terms of SD**	**ERA5 (JRA55)** **Maximum temperature in °C**	**ERA5 (JRA55)** **SD at the time of the event in °C**	**Notes**
Southeast Asia	24-Apr-98	5.1 (3.7)	32.8 (33.0)	0.5 (0.7)	16 of 32 days from 10 April to 10 May exceed 4 SDs in ERA-5
Southern Brazil	16-Nov-85	4.3 (4.1)	36.5 (36.4)	1.9 (2.0)	
USA: Alabama, Florida, Georgia, and Mississippi	13-Jul-80	4.3 (3.9)	38.4 (37.0)	1.7 (1.6)	
USA: southern Alaska	07-Jul-19	3.9 (4.2)	23.8 (24.2)	2.4 (2.5)	3 days (6–8 June) exceed 4 SDs in JRA-55
Southwestern Peru	24-Jan-16	4.2 (3.7)	23.0 (24.4)	0.9 (1.0)	
Canada: Alberta	30-Jun-21	4.1 (3.9)	36.0 (34.3)	3.4 (3.5)	Neighboring region, northern British Columbia, has magnitude of 4.01 on 29-Jun-21 in JRA-55
Southeast Brazil	07-Oct-20	3.7 (4.1)	36.7 (37.4)	1.9 (2.0)	
Canada: Yukon	14-Jun-69	3.9 (4.1)	27.3 (28.1)	3.2 (3.5)	

Four of the top events have occurred in North America, and globally, three have occurred within the past 3 years. Temporal clustering may occur because of decadal trends, but as we use a rolling climatology in this case, it does not indicate an increase in extremes. In ERA5, the Southeast Asian event in 1998 shows the greatest magnitude in terms of SD, and it spans a much longer time period (the event has a smaller magnitude and duration in JRA55). This event was likely driven by strong El Niño conditions. In 2016, a heat wave event in the same region, also driven by El Niño, was of higher absolute magnitude due to the increased influence of global warming (*40*).

We have shown that the western North America event of 2021 may have been caused by a combination of high pressure and dry conditions (e.g., Fig. 1), but it is well known that heat extremes in different parts of the world may be driven by other combinations of Earth system processes. There is evidence that the two events in Brazil (1985 and 2020) both could be drought-heat compound events as well, with the influence of a persistent high pressure system exacerbated by low soil moisture (*25*, *30*, *41*). Some other North American events may be associated with similar mechanisms. For example, the July 2019 event in Alaska saw record temperatures over several days peaking at 3.7 SDs from the mean. This event was driven by a persistent high-pressure system, and again accompanied by dry conditions.

As there is some evidence that human, animal, and ecological health correlate better with heat stress (the “felt” temperature) rather than with the dry bulb temperature measurements (*2*), we repeat our analysis using ERA-HEAT, a dataset of heat stress (see Materials and Methods and fig. S4). While the exact same time period cannot be compared because of the length of the datasets, our results broadly agree in between temperature and heat stress, at least in terms of the spatial patterns and years of occurrence. The absolute SD of each event does of course change, and the North American 2021 heat wave is again extreme in this dataset, reaching 3.75 SDs, the fourth highest region globally.

### Global heat extremes in the future

**Fig. 4.**
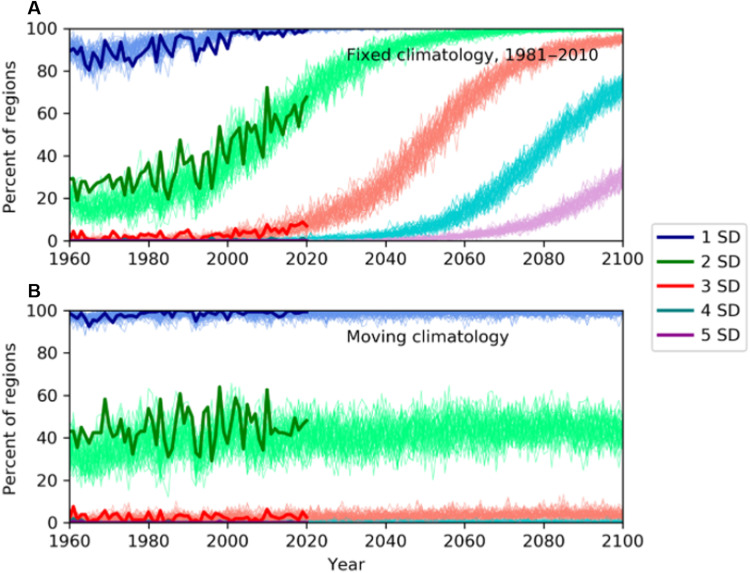
Historical and projected changes to temperature extremes. (**A**) Percentage of regions around the globe experiencing events each year above the specified thresholds of 1 to 5 SDs above the mean, calculated against the 1981–2010 historical baseline climatology for ERA5 (bold lines) and 50 ensemble members of the CanESM5, SSP585 (multiple thinner lines). (**B**) Same as (A), but calculated against a moving climatology of the decade before the year assessed to account for the climate change signal. As with Fig. 3, regions with poor agreement between ERA5 and JRA55 have been excluded in both ERA5 and CanESM5.

It has been suggested that the statistical distribution of heat extremes is changing as the world warms. Assuming a normal distribution, as the mean value increases, the proportion of events falling outside of 1, 2, or 3+ SDs would be expected to remain unchanged compared to the new distribution. Using the reanalysis data for each year, we calculate the number of regions experiencing events greater than 1, 2, or 3+ SDs calculated two ways—first using the 1981–2010 climatology and second based on the climatology of the previous decade. When calculated based on the 1981–2010 climatology, the number of events above each limit increases through time as the climate warms (Fig. 4A and fig. S4, A and C). On the basis of a running climatology of the previous decade the trends disappear, and the number of regions experiencing extremes above each limit is approximately stationary through time (Fig. 4B and fig. S4, B and D). We now see events above 4 SDs from the mean earlier in the record. At the time, these events would have seemed nearly as extreme as the heat wave observed in western North America in 2021—at least in terms of deviation from the normal conditions of that time. By their nature, very few events above 3 SDs will occur, making it difficult to quantify any change in these extremes using observational data; however, climate models can be used as an alternate approach to address this problem.

Large ensembles of model simulations are useful for investigating extreme events, as there are many times more data available than from the real world. We repeat the investigation of how the distribution of heat extremes is changing through time using two large ensembles, from CanESM5 and the sixth version of the Model for Interdisciplinary Research on Climate (MIROC6) (see Materials and Methods) (*42*). We include the MIROC6 ensemble to provide increased confidence in the results and as an indicator of the impact of the high climate sensitivity of the CanESM5 ensemble. The large ensemble sizes enable the extremes of the distribution to be investigated, as, by definition, more events at 3+ SDs will occur. Using ESMs also allows us to investigate projected changes out to 2100, beyond 2015, we use the SSP585 future scenario, which represents a high-emissions scenario.

We calculate the percentage of regions exceeding each SD threshold in each model ensemble member to make the results comparable to the reanalysis calculations. For the historical period, the reanalysis and model data show strong agreement. When calculating the extremes relative to the 1981–2010 climatology, the percentage of extremes above each threshold continues to increase into the future as the world continues to warm. For CanESM5, by 2100, all regions are seeing events above 2 SDs from the mean annually, and over 20% of regions experience extremes outside of 5 SDs from the mean. In MIROC6, the end-of-century values are lower, with 20% showing events 4 SDs from the mean. The lower climate sensitivity of this model leads to less warming in the future projection but, importantly, the trends are consistent with CanESM5. When repeating the calculations using a rolling mean, the model data show no increase in the proportion of events above each threshold (Fig. 4B and fig. S4, B and D). MIROC6 shows a larger proportion of events above each threshold, which is likely due to higher variance in the model (*42*). This suggests that extreme events will continue increasing in magnitude at the same rate as the mean shift.

## DISCUSSION

The temperatures observed during the western North America event of year 2021 are unprecedented in records from 1950 to present day for that location. We show evidence that a combination of high pressure and dry conditions may have contributed to the extreme heat conditions, a mechanism supported by studies of other heat wave events (*25*, *29*, *30*).

Quantifying the chance of extreme events is limited by the length of the observational record, but by using a model ensemble, we can assess a larger sample of meteorologically plausible events. The ensemble allows us to sample events more extreme than have been observed—and also more extreme relative to the model climatology. One role of climate models is to inform policymakers of levels of heat extremes expected at projected future atmospheric greenhouse gas concentrations. CanESM5 has a high climate sensitivity compared to other ESMs (*32*, *43*), leading to greater projected temperatures in future scenarios than other ESMs. The source of the higher climate sensitivity is thought to be changes in climate feedbacks, possibly associated with cloud and surface albedo feedbacks. It has been argued that we should be preparing for these “worst-case” scenarios, as the high climate sensitivity could be an accurate representation of the real world (*44*). If the real world is best described by ESMs with a high temperature response, this is of particular concern in the context of the heat-health problem (*45*). We assess a second large ensemble from MIROC6, a model with a low climate sensitivity. Similar trends are found, adding confidence to our conclusions (fig. S5).

We fit a GEV (generalized extreme value) distribution to model data to assess the chance of a simulated event as great as the one observed during June 2021 in western North America in terms of the model climatology. Previous studies have shown that the observed event lies outside the fit of reanalysis data (*27*). We find that the model fit can capture this extreme event, although as has been seen for the reanalysis product the most extreme events lie to the left of the GEV curve, appearing more frequent than GEV would suggest. Further investigation of global events using extreme value analysis would be valuable, such as testing other regions to assess whether other extremes are beyond the observed distributions and for other large ensembles.

The magnitude of extremes expressed in terms of SD from background climatology is an important indicator. In some cases, extremes that have a small absolute magnitude may have greater influence than larger extremes, because the region in which they occur is just not used to this temperature variability. Both human behavior and natural ecosystems will likely have adapted to levels of background variability, and so extremes of many SDs from the mean can be devastating. Generally, variability in daily temperatures is greatest in the extratropics and smaller in tropical regions.

Critically, we show that, in both reanalysis and model data, in regions across the globe, heat extremes are not becoming any more extreme relative to climatology for the previous decade. Hence, the observed and projected increases in extreme event occurrence are almost always caused simply by changes to the mean background state. This finding agrees with, and adds confidence to, the conclusions of the Intergovernmental Panel on Climate Change (IPCC) Sixth Assessment Report (AR6) (*1*), that there are observed changes in the extremes, but these shifts in temperature extremes are explainable by shifts in the mean.

Although our study identifies major extreme events, it is important to note regions with a low greatest historical extreme. If the statistical distribution of daily maximum temperature is broadly the same across the globe (after normalization by its SD), some regions have not—just by chance—experienced large extremes in the reanalysis period. These regions may be less prepared for high temperatures, as there has not yet been need to adapt. Our analysis shows that parts of India, Australia, and central Africa show no events more than 3 SDs from the mean. Statistically, such an event should be expected once every ~30 years.

A further caveat of our analysis is that the events of Table 1 are not a definitive list of the most extreme events. Small changes to methodology, such as the temporal resolution or level of agreement between datasets, could change the events identified, or their order in terms of most extreme. For our particular methodology, with heat wave magnitude described in terms of SDs away from the mean, the western North America event of 2021 is not unprecedented when compared with other heat waves around the world, although it is still remarkable. We acknowledge that there remains debate surrounding the geographical definition of extreme events, and different scale regions would alter our results. Assessing globally, we use predefined regions (*35*). However, there are other options available, e.g., artificial intelligence pattern–based algorithms to self-select extreme events (*46*). The most recent AR6 IPCC report provides a new set of regions (*1*). Assessing extremes at a higher spatial resolution may allow localized hot spots to be identified, but the internal variability of the climate system implies that the specific location of extremes will vary between years.

While we are confident of the broad applicability of our analysis, we hope that our approach will encourage further regional investigations of other historical heat extremes, or new ones as they occur. We only investigate in detail the shape of the daily maximum temperature distribution for the western North America region; if data from some regions are more skewed than others, the measure of SD from mean will be less meaningful. Any time-evolving changes to the shape, scaling, and asymmetries of temperature distributions require a more complete analysis regionally. These expanded analyses may include other metrics of extremes beyond maximum daily temperature, such as mean seasonal temperature, or 5-day means. Noting our finding of very dry conditions during the 2021 heat wave, further work could assess the conditional nature of heat extremes. Such an approach may characterize precipitation, drought, and how they could drive atmospheric conditions (*47*). There is much scope for more use of large ensembles by a range of ESMs in assessing extremes, in both the current climate and the future. Large ensembles of ESMs, including recent multimodel large ensembles (*33*), provide multiple plausible extremes for which we can analyze the mechanisms and dynamical limits to the extremes. We appeal to the ESM community to perform these ensembles, where computational power can allow this.

In conclusion, the western North America heat wave of June 2021 was an exceptional event. For that region, the extreme event was unprecedented in the observational record in terms of absolute magnitude and heat stress level. By 2080, for CanESM5 under SSP585, such an event would have a 1-in-6 chance of occurring each year. However, we have also shown that a small number of other heat events of a similar magnitude, as compared to the local variability, have occurred in recent decades in other parts of the world. We show that extremes appear to increase in line with changes to the mean state of the distribution of the climate, and projected increase in extremes aligns with projected warming.

## MATERIALS AND METHODS

### Experimental design

#### Reanalysis and observation data

We use reanalysis data for our study. These datasets provide spatially complete gridded climate data by combining observational records with data from forecasting models and data assimilation systems used to fill gaps where direct observations are unavailable. We use ERA5 and JRA55 within our analysis (*36*–*38*). These datasets allow us to make a global assessment of extremes. Data are available from 1950 to present (ERA5) and 1958 to present (JRA55). The use of two different reanalysis datasets allows us to have increased confidence in our results.

We use observational data to verify the most extreme events. Global Historical Climatology Network daily (GHCNd) data are used (*48*). This is a database of daily climate summaries from land surface stations globally. Over 100,000 stations are included across 180 countries, and all stations undergo quality assurance. The closest station to the center point of the region, which has at least 10 years of data covering the extreme event, is used to verify the event.

To assess heat stress, we use ERA5-HEAT (*49*). This ECMWF product is derived from ERA5 and provides a global spatially gridded dataset of a measure of human heat stress, from 1979 to present. The Universal Thermal Climate Index (UTCI) is used as the measure of human heat stress, and it combines air temperature, wind, radiation, and humidity to give a biometeorological index for assessment of human health impacts of climatic conditions (*50*).

#### Model data

Two large ensembles, of CanESM5 and MIROC6, are used to assess future changes in the extremes (*32*, *42*). CanESM5 combines an atmospheric general circulation model with ~2.8° resolution, an ocean general circulation model with ~1° resolution, a sea-ice model, a land surface scheme, and explicit land and ocean carbon cycle models. MIROC6 combines an atmospheric model with a ~1.4° resolution, with land, sea ice, and ocean model components (*42*). Both models have a relatively coarse resolution compared to other CMIP6 models, which allows a large ensemble to be produced more easily. Generally, as the resolution of models is increased, their performance improves (*51*), although for daily maximum temperature specifically it has been found that, comparing models at their native resolution, the CMIP6 ensemble does not improve performance compared to CMIP5 (*52*). For both models, we use 50-member ensembles, with historical simulations (1950–2014) and future projections (2015–2100). The future projections follow the experimental design of the Scenario Model Intercomparison Project of CMIP6 (*33*), using the SSP-RCP (Shared Socioeconomic Pathway–Representative Concentration Pathway) scenarios (*34*) SSP126, SSP245, and SSP585. CanESM5 has a high equilibrium climate sensitivity of 5.6 K, whereas MIROC6 is at the lower end at 2.7 K.

For CanESM5, we assess the model performance over the region of the western North America heat wave of 2021. The region used is a box, 45°N to 52°N, 237°E to 241°E (*25*). The model data are compared to reanalysis data from ERA5, for daily maximum temperature for boreal summer (JJA) over the period 1981–2010. We find that the model is broadly consistent with observations (fig. S1); however, the observed mean and SD do not lie within the range of individual ensemble members. Because of this, we assess the model against itself when evaluating the chance of the occurrence of observed extreme of June 2021. Using the model climatology to calculate the magnitude of an event 3.6 model SDs from the model mean, we find that an extreme equivalent to the observed June 2021 events is 40.1°C for CanESM5.

Extreme value theorem can be used to assess the chance of a specific extreme event. We apply a GEV distribution to the annual maximum of the daily maximum present-day model data, 2015–2024 (fig. S2). We use this to assess the chance of a model event of 40.1°C or greater in the present day.

We assess the chance of the daily maximum temperature exceeding the magnitude of the observed record event in June 2021, over the region, on any day in JJA, which is 40.1°C based on the model climatology (Fig. 2). We calculate this over rolling 10-year periods for the three future scenarios. We assume that every day is independent, with an equal chance of exceeding the record—thus calculating the chance of exceeding the record on any given JJA day within the 10-year period. To assess the uncertainty, we plot the range of the values for each individual ensemble member.

#### Regions

When assessing climatic extremes, spatial scale is important. Station data can provide information of localized extremes, likely unable to be modeled by global scale ESMs. Increasing the spatial scale will dampen the magnitude of an extreme—a balance must be reached. National boundaries could be used, but countries vary vastly in size. A study defined five sets of regions designed for assessing climatic extremes at different target sizes from 0.1 to 10 Mm^2^ (*35*). Regions are based on political and economic divides and based on impacts, aligned with decision-making and disaster response rather than climatology. Similar sets of regions have been used to assess daily extremes already, for example, daily extremes, in reanalysis datasets using 2-Mm^2^-scale regions (*52*). Using a scale of 2 Mm^2^ or smaller allows extremes caused by mid-latitude synoptic scale weather systems to be incorporated (*53*). Throughout this study, we will use the predefined 0.5-Mm^2^ regions; this scale corresponds to a diameter of ~800 km (*35*). There are 237 regions in the dataset, but because of the way the regions are defined, using political boundaries based on impacts, some areas of the world are excluded. The main areas missed are Armenia, the Balkans, Bangladesh, most Caribbean islands, Belarus, Georgia, Nepal, New Zealand and most other Pacific Islands, North Korea, and Sri Lanka. Our choice of spatial regions larger than the dataset resolutions is supported by earlier studies; it has been found that when considering only the single gridbox scale, robust statements on the expected changes to extremes are difficult to make (*54*).

When investigating the western North America heat wave, we use the region used in the World Weather Attribution study (*28*). The area is 45°N to 52°N, 119°W to 123°W. This was chosen to include the cities of Portland, Seattle, and Vancouver but exclude the less populated regions to the west and east, which also experienced extreme temperatures. The region was defined to focus on the human impacts of the event.

#### Calculating the extremes index

The daily maximum temperature data from 1950 to 2021 are analyzed. First, they are subdivided into 237 regions (*35*), and then the regional mean is calculated. A daily extreme index is calculated, which is a measure of the temperature in terms of SD.

Daily extreme index=(Daily maximum temperature−mean daily maximum temperature)/Standard deviation

The month with the greatest mean temperature is found over the period 1981–2010, and the 3 months centered on that are used to calculate the mean daily maximum temperature and SD. Using a 3-month window will lead to the inclusion of seasonality in some regions, but in the global assessment, the hottest climatological month shows low variability, which would cause anomalously large extremes to be identified if using a 1-month window. The daily extreme index is then calculated every day of the year.

The index is calculated with two different climatologies. We assess the daily extremes based on the climatology of 1981–2010 to allow us to investigate how the extremes are varying through time. We also assess using the mean daily maximum temperature and SD calculated based on the 10 years preceding. This method ensures that the extremes are assessed relative to the climate of that period, preventing earlier extremes from becoming ignored. For both methods, we identify the value and year of the maximum daily extreme index.

To ensure reliability of results, we use two different reanalysis datasets, ERA5 and JRA55, eliminating regions where there is disagreement between the datasets. As JRA55 is available only from 1958, we can only assess from 1968 (allowing a 10-year averaging period). First, any regions that disagree on the mean warmest month by more than one calendar month over the period 1981–2010 are removed. Second, we calculate the value and year of the maximum daily extreme index in ERA5. We check that the year of the ERA5 maximum is in the top 5 years for the region for JRA55, removing the regions where this is not the case. This leaves us with 158 regions in Figs. 3 and 4. The same set of regions is used for the model data in Fig. 4 and for ERA-HEAT in fig. S2. In Table 1, we list eight events that are consistent between the two reanalysis datasets, and are greater than 4 SDs in one dataset and greater than 3.5 SDs in the other.
