# Supplementary material for: The 2021 western North America heat wave among the most extreme events ever recorded globally
Source: Sci Adv. 2022 May 4;8(18):eabm6860. doi: 10.1126/sciadv.abm6860 (PMC9067932; doi:10.1126/sciadv.abm6860)
Supplement: Supplementary file 1 — Figs. S1 to S5 [file sciadv.abm6860_sm.pdf]

Science Advances
AAAS

Supplementary Materials for

The 2021 western North America heat wave among the most extreme events ever recorded globally

Vikki Thompson*, Alan T. Kennedy-Asser, Emily Vosper, Y. T. Eunice Lo,
Chris Huntingford, Oliver Andrews, Matthew Collins, Gabrielle C. Hegerl, Dann Mitchell

*Corresponding author. Email: vikki.thompson@bristol.ac.uk

Published 4 May 2022, *Sci. Adv.* **8**, eabm6860 (2022)
DOI: 10.1126/sciadv.abm6860

This PDF file includes:

Figs. S1 to S5

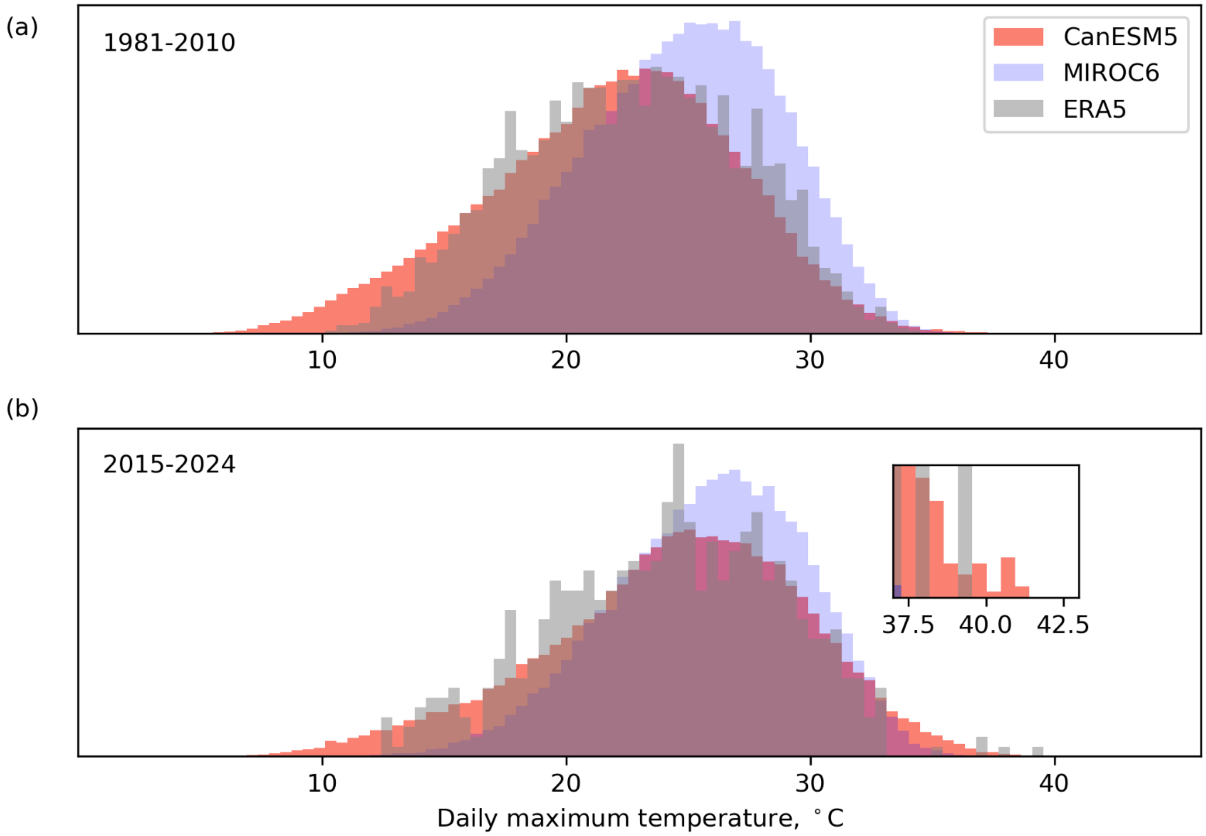

Fig. S1.

Consistency between ERA5, CanESM5, and MIROC6 for western North America. (**A**) Histogram of June, July, and August daily maximum temperatures, in °C, from 1981-2010, for the region 45°N-52°N, 119°W-123°W for reanalysis data from 30 years of ERA5 (grey) and model data from 50 ensemble members (1500 years) of CanESM5 (red) and MIROC6 (blue). (**B**) As (A) for 2015-2024, with a subplot showing the tail of the distributions magnified to highlight the unprecedented modelled temperatures in CanESM5.

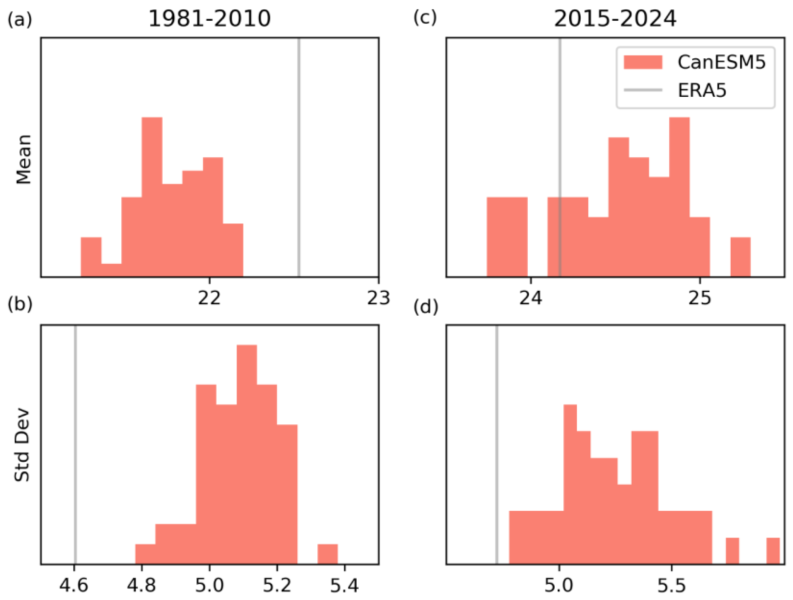

Fig. S2.

Comparing the statistics of ERA5 and CanESM5 data for western North America. Histograms of the mean and standard deviation of the June, July, and August daily maximum temperature, in °C, for the 50 ensemble members of CanESM5 for the western North America region (45°N-52°N, 119°W-123°W), with the single ERA5 value as a vertical grey line. (**A**) shows the mean from 1981-2010, (**B**) shows standard deviation from 1981-2010, (**C**) shows the mean from 2015-2024 (ERA5 2015-2021), (**D**) shows standard deviation from 2015-2024 (ERA5 2015-2021).

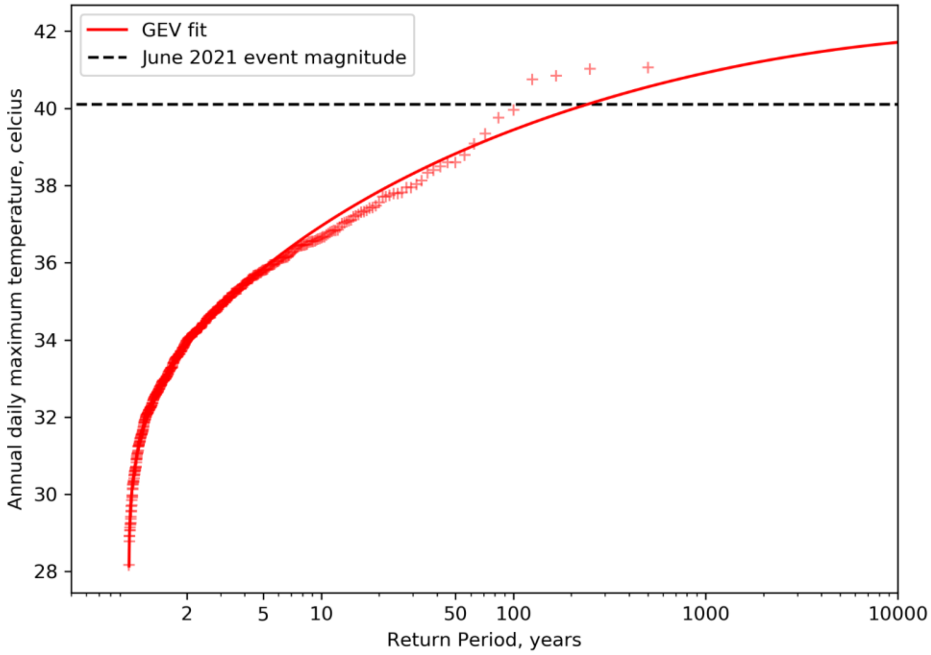

Fig. S3.

Extreme value analysis of model data representing the present-day. This is a return period plot of annual maximum daily temperature, °C, for years in period 2015-2024 inclusive, with values from 50 ensemble member calculations by the CanESM5 climate model, and for the western North America region (45°N-52°N, 119°W-123°W) region. Crosses indicate the model values, and the curve indicates the fitted GEV distribution. The dashed black line shows the magnitude of the observed June 2021, adjusted for the model climatology.

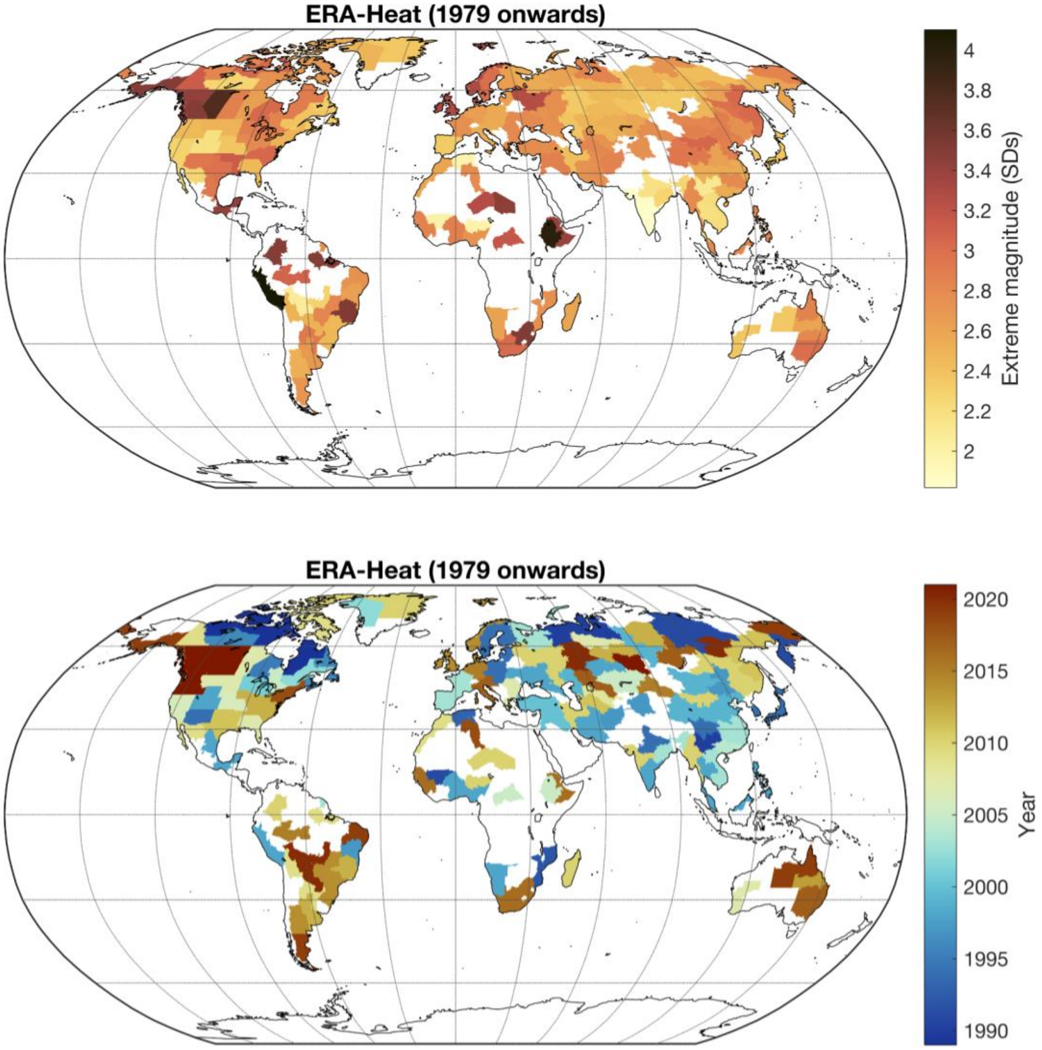

Fig. S4.

Global map of the most extreme heat stress events. Heat stress index taken from ERA5-HEAT, January 1979 - August 2021. The values are expressed in terms of how many standard deviations away from the mean the most extreme heat stress day was (see Methods). The regions are taken from Stone, 2019 (33). (A) Shows the value of the greatest historic extreme in each region. (B) Shows the year of the greatest historic extreme. Regions are excluded where there is poor agreement between daily maximum temperature for ERA5 and JRA55 reanalyses. N.B. Different dates to Fig.3 due to current data availability of ERA5-HEAT.

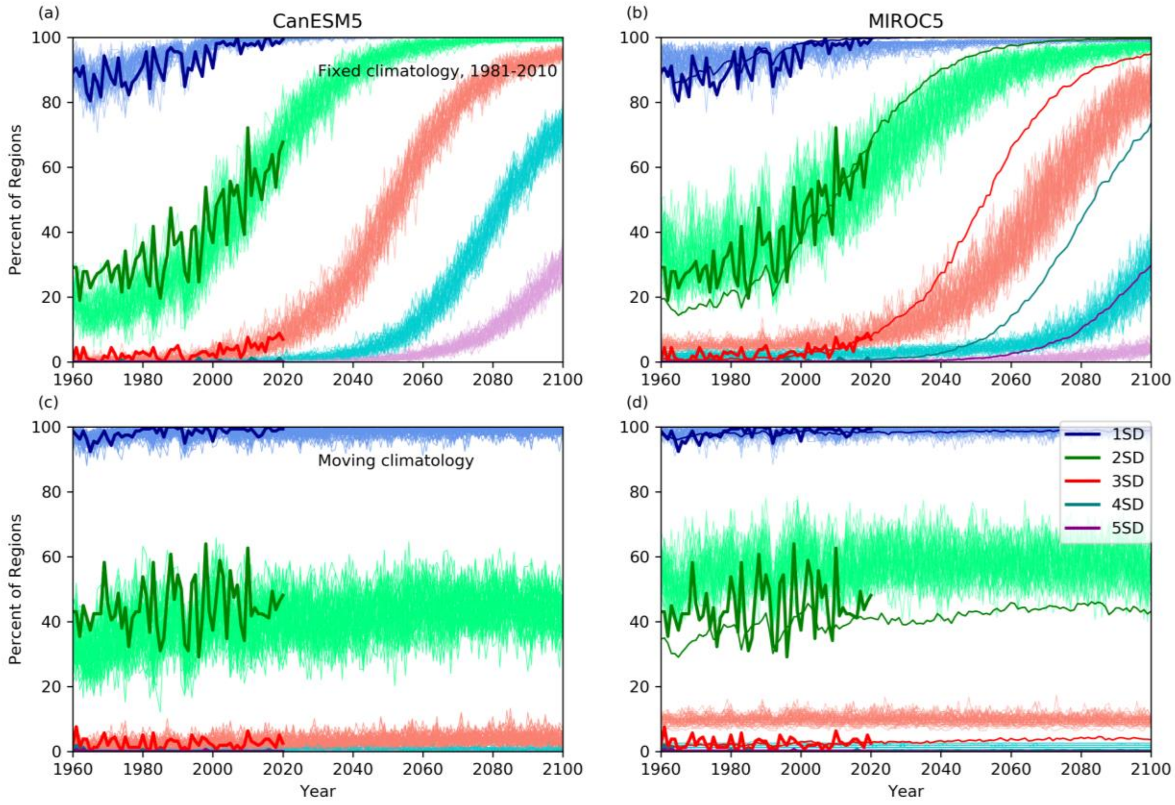

Fig. S5.

Historical and projected changes to temperature extremes from two large model ensembles. (**A**) same as Fig4a, shows the percentage of regions around the globe experiencing events each year above the specified thresholds of one to five standard deviations above the mean, calculated against the 1981-2010 historical baseline climatology for ERA5 (bold lines) and 50 ensemble members of the CanESM5 (multiple thinner lines), (**B**) same as Fig.4b, shows the same as (**A**), but calculated against a moving climatology of the decade prior to the year assessed to account for the climate change signal. As with Figure 3, regions with poor agreement between ERA5 and JRA55 have been excluded in both ERA5 and CanESM5. (**C**) similar to (**A**) but showing 50 ensemble members from MIROC6 (thinnest lines), with the mean of the CanESM5 ensemble also shown (thin lines). (**D**) similar to (**B**) but for the MIROC5 50 member ensemble.
